# Differential pathogenesis and antiviral treatment outcomes of MPXV clade Ib and clade IIb in a BALB/c mouse model

**DOI:** 10.1080/22221751.2026.2724633

**Published:** 2026-09-16

**Authors:** Yong Feng, Chenguang Shen, Penghui Jia, Bo Peng, Wei Yang, Shiman Chen, Yun Peng, Xiaowen Liang, Shengjie Zhang, Fangfang Chang, Yuanlong Lin, Mingxia Zhang, Yingxia Liu, Baisheng Li, Fuxiang Wang, Yang Yang

**Affiliations:** a Shenzhen Key Laboratory of Pathogen and Immunity, Shenzhen Third People's Hospital, Second Affiliated Hospital, School of Medicine, Southern University of Science and Technology, Shenzhen, People’s Republic of China; b National Clinical Research Center for Infectious Disease, Shenzhen, People’s Republic of China; c BSL-3 Laboratory (Guangdong), Guangdong Provincial Key Laboratory of Tropical Disease Research, School of Public Health, Key Laboratory of Infectious Diseases Research in South China, State Key Laboratory of Multi-Organ Injury Prevention and Treatment, Southern Medical University, Ministry of Education, Guangzhou, People’s Republic of China; d Institute of Microbiology, Center for Disease Control and Prevention of Guangdong Province, Guangzhou, People’s Republic of China; e Pathogen Detection for Emerging Infectious Disease Response, Guangdong Provincial Key Laboratory of Pathogen Detection for Emerging Infectious Disease Response, Guangzhou, People’s Republic of China; f Shenzhen Guangming District Center for Disease Control and Prevention, Shenzhen, People’s Republic of China; g Intelligent Tracking and Forecasting for Infectious Disease, NHC Key Laboratory of Medical Virology and Viral Diseases, National Institute for Viral Disease Control and Prevention, Key-Laboratory of Intelligent Tracking and Forecasting for Infectious Disease, Beijing, People’s Republic of China

**Keywords:** MPXV, Clade Ib, BALB/c mouse model, pathogenesis, antiviral efficacy

## Abstract

The global emergence of monkeypox virus (MPXV) clade IIb since 2022, together with the recent spread of clade Ib, underscores the continuing public health threat posed by MPXV. Clinical and epidemiological observations suggest that clade Ib infection may differ from clade IIb infection, but the biological basis of these differences remains insufficiently defined. In this study, we established an intranasal BALB/c mouse model of MPXV clade Ib infection and compared its pathogenicity with that of clade IIb. Clade Ib caused lethal disease at an inoculation dose approximately ten-fold lower than that required for clade IIb. Compared with clade IIb, clade Ib infection showed faster disease progression, higher pulmonary viral burden at later stages of infection, and more severe lung pathology. Single-cell transcriptome and cytokine response analyses further uncovered clade-specific differences in pulmonary immune cell landscapes, the distribution of MPXV-positive cells, and the expression patterns of key inflammatory mediators at specific time points. Antiviral evaluation showed that tecovirimat and cidofovir were active against both clades, although a higher dose of cidofovir was required to achieve protection in the clade Ib infection model. Together, these findings establish a susceptible BALB/c mouse model for MPXV clade Ib and provide experimental evidence for clade-dependent differences in MPXV pathogenicity and antiviral treatment outcomes.

## Introduction

Mpox (formerly monkeypox) is a zoonotic disease caused by the monkeypox virus (MPXV), which belongs to the genus Orthopoxvirus [1]. MPXV was first identified in 1958 in research monkeys imported from Singapore [2], and the first human case was confirmed in 1970 in the Democratic Republic of the Congo (DRC) [3]. Human infection may lead to symptoms such as fever, headache, lymphadenopathy, and rash, typically lasting 2–4 weeks. Although most patients recover spontaneously, pregnant women, children, and immunocompromised individuals are at higher risk of severe complications or death [4,5]. Traditionally, MPXV was transmitted to humans mainly through rodent reservoirs, with limited subsequent human-to-human spread [6]. However, recent studies indicate that human-to-human transmission may occur through close physical contact, sexual contact, exposure to respiratory secretions, contaminated materials, and healthcare-associated exposure [7]. Orthopoxviruses, including VACV and MPXV, can infect susceptible hosts through mucosal surfaces, including the respiratory tract, under experimental or exposure-related conditions [8, 9]. In human mpox, viral DNA has been detected in saliva and oropharyngeal samples, and mucosal lesions such as enanthema and oropharyngeal ulceration may contribute to viral shedding during infection [3,10,11].

MPXV is classified into two major clades, clade I (Central African, case fatality rate up to 10%) and clade II (West African, case fatality rate around 2%) [12]. Since 2022, successive outbreaks have revealed striking differences in their epidemiology and pathogenicity [13]. The 2022 global outbreak was driven by clade IIb, a sub-lineage of the West African clade, which spread to more than 100 countries with over 80,000 confirmed cases [14]. Transmission was mainly associated with high-risk sexual contact, particularly among men who have sex with men (MSM), while respiratory spread was rarely observed, and the overall case fatality rate remained low at approximately 0.22% [15]. In contrast, clade Ib, first identified in South Kivu Province of the Democratic Republic of the Congo in late 2023, has subsequently spread to multiple African countries and has been detected in several non-African countries, including Sweden, Thailand, and China [16–20]. Unlike the 2022 global outbreak caused by clade IIb, clade Ib outbreaks have affected a substantial proportion of children in some regions and have been associated with more severe disease, sustained human-to-human transmission, and reported case fatality rates of 0.3%–1.4% according to WHO reports [21,22]. These observations indicate important epidemiological and clinical differences between clade Ib and clade IIb and underscore the need to investigate the biological determinants that may contribute to the emergence and spread of clade Ib.

The establishment of animal models that recapitulate human clinical manifestations is crucial for elucidating MPXV pathogenesis and the biological characteristics of emerging clades [23]. Existing MPXV animal models include non-human primates (NHPs), wild rodents, and other rodent species. NHPs are considered a fundamental model for studying MPXV and other emerging pathogens due to their high immunological and anatomical similarity to humans. They can reproduce systemic infection and pustular rash similar to human disease; however, their use is limited by ethical concerns, high costs, and operational complexity [9,24,25]. Wild rodents such as ground squirrels (Marmota bobak) and African dormice are also highly susceptible to MPXV, yet their complex breeding requirements, low reproduction rates, and limited commercial reagents make them difficult to use widely [26–28]. In contrast, rabbits are more cost-effective, easier to handle, and available as genetically well-defined populations, but their immunological differences from humans limit their applicability [29,30]. Immunodeficient mice and certain inbred strains (e.g. CAST/EiJ, MOLF/EiJ, and PERA/EiJ) are highly sensitive to MPXV. Clade Ia can cause severe or fatal disease in these models, while clade IIb shows weaker pathogenicity [31–35]. However, such models face challenges including immune abnormalities, limited availability, and difficulties in breeding and handling, which restrict their use in MPXV pathogenesis research and the evaluation of drugs and vaccines.

BALB/c mice serve as an important alternative model for orthopoxvirus research due to the low cost, easy availability, and well-established experimental tools. Previous studies have shown that vaccinia virus (VACV) and MPXV clade IIb can cause severe or lethal infection in BALB/c mice, and this model has been widely used for preliminary evaluation of anti-poxvirus drugs, neutralizing antibodies, and vaccines [36–40]. Currently, there is still a lack of a reliable animal model for MPXV clade Ib, and the pathogenicity differences between clade Ib and clade IIb have not been systematically elucidated.

In this study, we established an intranasal BALB/c mouse infection model for MPXV clade Ib and systematically compared its pathogenicity with that of clade IIb. This model provides a useful platform for studying clade-dependent differences in MPXV virulence, pulmonary pathology, host immune responses, and antiviral efficacy.

## Results

### MPXV clade Ib causes more severe clinical signs than MPXV clade IIb in intranasally infected BALB/c mice

To establish the infection model of clade Ib in BALB/c mice, we first infected the mice with an empirical dose of 2 × 10^5^ PFU based on a previous report showing that clade IIb infection at this dose resulted in weight loss and mortality in BALB/c mice [39]. Following intranasal inoculation with clade Ib, mice were monitored daily for clinical signs. Infected animals developed ruffled fur from 3 days post infection (dpi), progressing to hunched posture and reduced food and water intake. Notably, one mouse exhibited ∼30% body weight loss by 4 dpi, and all animals succumbed by 5 dpi (Figure S1).

**Figure 1.**
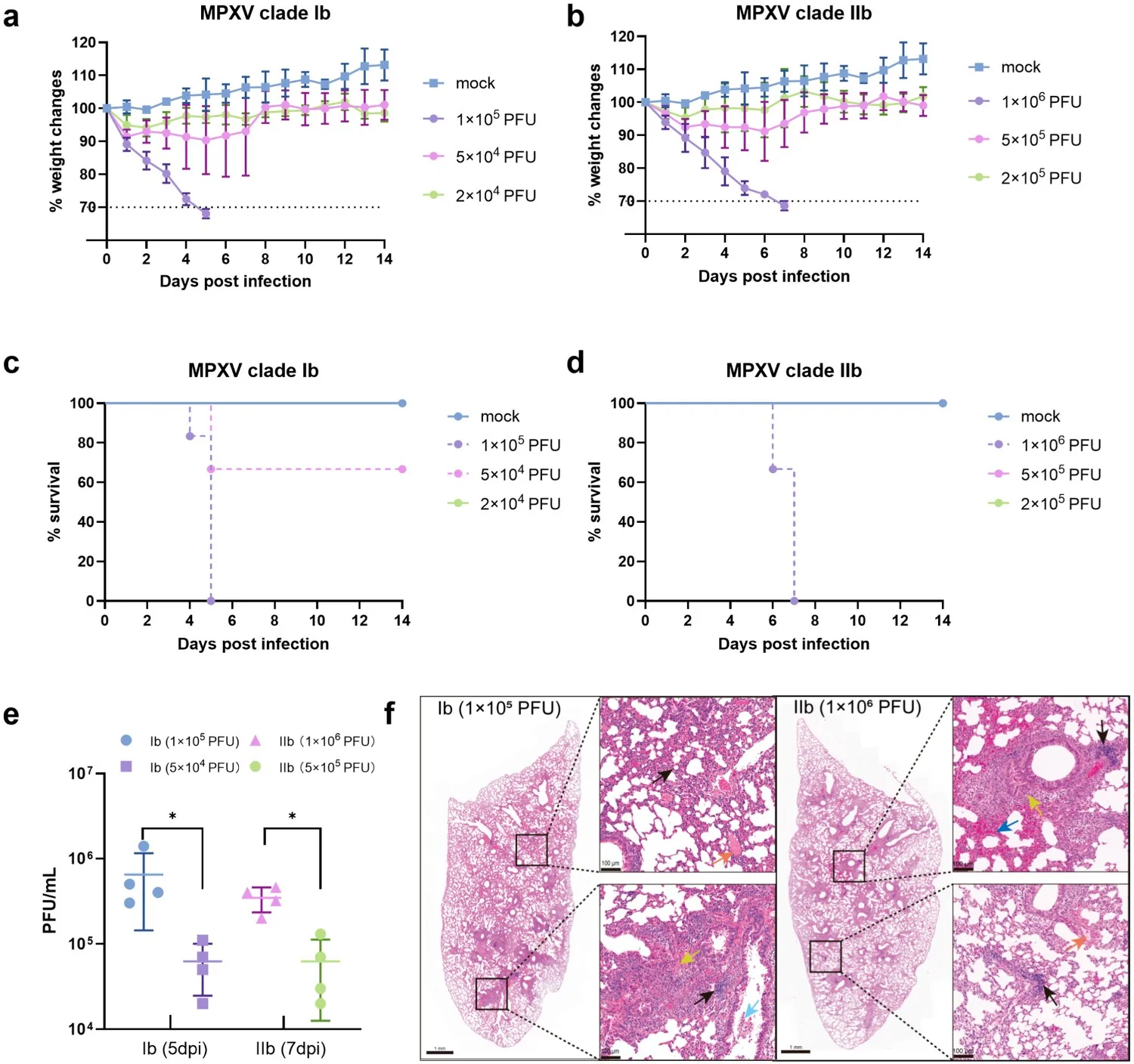
MPXV clade Ib causes lethal disease at a lower dose than clade IIb following intranasal infection of BALB/c mice. (a–d) Intranasal infection of BALB/c mice with different doses of MPXV clade Ib and clade IIb. (a, c) BALB/c mice were intranasally inoculated with MPXV clade Ib at doses of 1 × 10⁵ PFU, 5 × 10⁴ PFU, or 2 × 10⁴ PFU (n = 6 per dose group). (b, d) BALB/c mice were intranasally inoculated with MPXV clade IIb at doses of 1 × 10⁶ PFU, 5 × 10⁵ PFU, or 2 × 10⁵ PFU (n = 6 per dose group). Body weight (a, b) and survival (c, d) were monitored daily. (e) BALB/c mice (n = 6) were intranasally infected with different doses of MPXV clade Ib (5 × 10⁴ PFU or 1 × 10⁵ PFU) or clade IIb (5 × 10⁵ PFU or 1 × 10⁶ PFU). Infectious virus in the lungs was quantified by plaque assay at 5 or 7 dpi, respectively. (f) H&E staining of lung sections from MPXV clade Ib (1 × 10⁵ PFU) at 5 dpi or clade IIb (1 × 10⁶ PFU) at 7 dpi. Black arrow: Lymphocytic and granulocytic infiltration. Light blue arrow: Necrotic cellular debris. Yellow arrow: Fibroplasia. Blue arrow: Haemorrhage. Orange arrow: Eosinophils. Scale bars for H&E staining: 1 mm (overview), 100 μm (magnified view). Data are presented as mean ± SD, *P < 0.05 by the Mann–Whitney test.

We next performed a dose–response experiment in female BALB/c mice (n = 6) with graded doses to further characterize the pathogenic profile of clade Ib (2 × 10^4^, 5 × 10^4^, and 1 × 10^5^ PFU), and also a systematic comparison with clade IIb (2 × 10^5^, 5 × 10^5^, and 1 × 10^6^ PFU). Both groups developed severe respiratory and systemic disease, including dyspnea, coughing, inactivity, lethargy, poor coat condition, hunching, and decreased responsiveness at high doses (clade Ib, 1 × 10⁵ PFU; clade IIb, 1 × 10⁶ PFU). Notably, clade Ib induced rapid weight loss from 3 dpi (Figure 1a), with one mouse euthanized at 4 dpi due to >30% weight loss and the remainder succumbing by 5 dpi (Figure 1c). By comparison, clade IIb infection progressed more slowly with two mice euthanized at 6 dpi, and the rest died by 7 dpi (Figure 1b, d). At medium inocula, clade Ib (5 × 10⁴ PFU) caused lethal disease in 2 of 6 mice by 5 dpi, whereas clade IIb (5 × 10⁵ PFU) produced no fatalities. At the lowest doses tested (clade Ib, 2 × 10⁴ PFU; clade IIb, 2 × 10⁵ PFU), neither infection was lethal, and body weights recovered after transient loss. Based on the mortality observed across the tested dose range, the median lethal dose (LD_50_) was estimated to be approximately 5.9 × 10⁴ PFU for clade Ib and 7.1 × 10⁵ PFU for clade IIb following intranasal infection of BALB/c mice.

Moreover, the lungs of the infected mice were collected (clade Ib at 5 dpi and clade IIb at 7 dpi), and the viral titers as well as histopathological changes were analyzed. Infectious virus was detected in lung tissues from mice in the lethal and medium-dose groups, whereas no infectious virus was recovered from the low-dose groups (Figure 1e). For infectious titers for the clade Ib-infected mice, high viral titers with a median value of 6 × 10⁵ PFU were observed in the lethal dose group (1 × 10⁵ PFU), followed by a median value of 6.3 × 10^4^ PFU for the medium-dose group (5 × 10^4^ PFU), while no infectious titers were measured for the low-dose group (2 × 10^4^ PFU). Comparatively, the mice infected with clade IIb at a lethal dose (1 × 10⁶ PFU) reached a median lung viral titer of only 3 × 10⁵ PFU at 7 dpi, followed by a median value of 6.2 × 10^4^ PFU for the medium-dose group (5 × 10^5^ PFU), and also no infectious titers were measured for the low-dose group (2 × 10^5^ PFU). Histopathological analyses of lung tissues from the lethal groups revealed comparable pulmonary damage for both clade Ib and IIb challenge, including granulocyte and lymphocyte infiltration, alveolar wall thickening, widened alveolar septa, and epithelial oedema (Figure 1f).

### Comparative tissue distribution, lung replication, and respiratory tract dynamics of MPXV clade Ib and clade IIb in BALB/c mice

**Figure 2.**
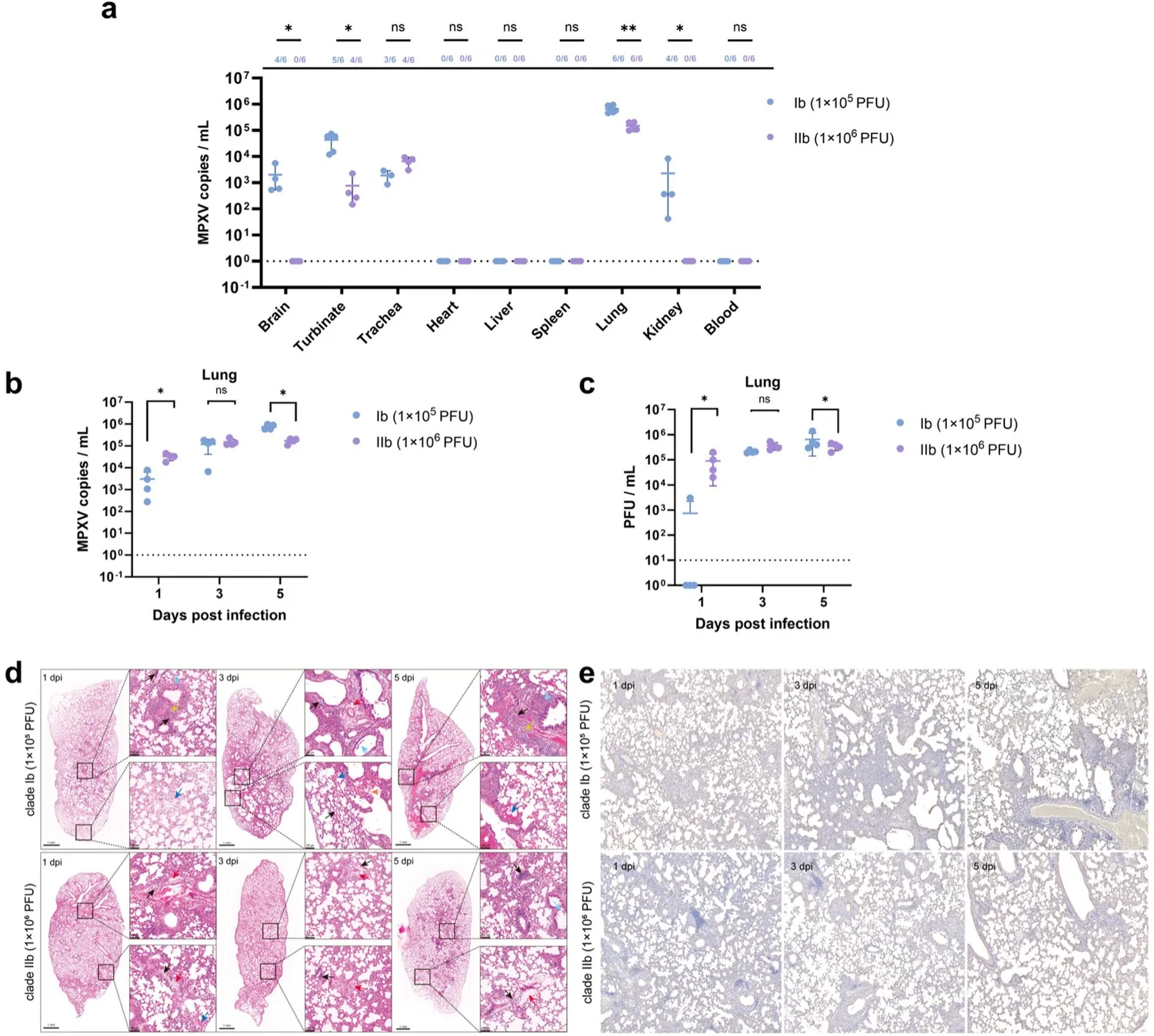
MPXV clade Ib shows higher pulmonary burden at later infection stages and earlier lung pathology than clade IIb in BALB/c mice. (a) Viral loads of MPXV clade Ib and clade IIb in mouse tissues and blood by qPCR at 5 dpi. Numbers above the scatter diagram denote the positivity rate of samples. (b, c) BALB/c mice (n = 4) were intranasally infected with 1 × 10⁵ PFU of MPXV clade Ib and 1 × 10⁶ PFU of clade IIb. Lung tissues were collected at 1, 3, and 5 dpi, and viral load was quantified using plaque assay for infectious particles and qPCR for genome copies. Data are presented as mean ± SD, *P < 0.05, **P < 0.01 by the Mann–Whitney test. Representative H&E-stained (d) and IHC (e) lung sections from mice infected with MPXV clade Ib (1 × 10^5^ PFU) or IIb (1 × 10^6^ PFU) at 1, 3, and 5 dpi. Black arrow: Lymphocytic and granulocytic infiltration. Light blue arrow: Necrotic cellular debris. Blue arrow: Haemorrhage. Yellow arrow: Fibroplasia. Red arrow: Perivascular oedema. Orange arrow: Eosinophils. Scale bars for H&E staining: 1 mm (overview), 100 μm (magnified view). Brown staining indicates viral antigen. Scale bars: 0.5 mm.

Major organs were collected to assess tissue distribution of MPXV following intranasal infection with lethal challenge doses of clade Ib (1 × 10⁵ PFU) or clade IIb (1 × 10⁶ PFU). Viral DNA quantification by qPCR showed that clade Ib viral DNA was detected predominantly in respiratory tissues, including the turbinate, trachea, and lung, with additional low-level detection in the brain and kidney. In contrast, clade IIb viral DNA was mainly detected in respiratory tissues, including the turbinate, trachea, and lung (Figure 2a). These results indicate limited extrapulmonary detection of clade Ib viral DNA rather than widespread systemic dissemination. Low levels of viral DNA were also detected in oropharyngeal swabs from both clades at 5 dpi (Figure S2).

We next examined early lung replication dynamics at 1, 3, and 5 dpi following lethal-dose infection (n = 4). Viral DNA was detectable in the lungs of both groups at 1 dpi, with median values of 3 × 10³ copies/mL for clade Ib and 3 × 10⁴ copies/mL for clade IIb. At 3 dpi, lung viral DNA levels were comparable between the two groups. By 5 dpi, clade Ib showed higher viral genome levels than clade IIb (median: 7.5 × 10⁵ versus 1.7 × 10⁵ copies/mL) (Figure 2b). Plaque assays performed on the same samples showed a similar pattern of infectious virus production (Figure 2c).

To determine whether the lung replication phenotype could be reproduced in vitro, growth kinetics of clade Ib and clade IIb were examined in A549 human pulmonary epithelial cells and MRC-5 human lung fibroblasts. Cell-associated virus (CAV) and extracellular virus (EV) were quantified separately. In one-step growth assays, both clades produced comparable CAV and EV titers in A549 and MRC-5 cells at 48 h post-infection. In multi-step growth assays, the two clades also showed broadly similar replication kinetics in both cell lines, although clade Ib showed a modest trend toward higher EV production in A549 cells at later time points (Figure S3). These findings indicate that the higher clade Ib viral burden observed in mouse lungs was not fully recapitulated in these lung-derived cell culture models.

Histopathological examination revealed earlier and more severe lung lesions in clade Ib-infected mice than in clade IIb-infected mice. At 1 dpi, clade Ib-infected lungs showed focal bronchiolar and perivascular necrosis, cellular debris, connective tissue proliferation, lymphocyte and granulocyte infiltration, perivascular oedema, and alveolar haemorrhage. By 3 dpi, lesions progressed to widespread necrosis, extensive inflammatory infiltration, alveolar expansion, and pronounced oedema. At 5 dpi, multifocal destruction of bronchiolar and perivascular structures, haemorrhage, congestion, and chronic inflammation were observed. In contrast, clade IIb infection induced milder and slower-progressing pathology, with minimal inflammatory infiltration and alveolar septal thickening at 1 dpi, limited necrosis and moderate oedema at 3 dpi, and mild lesions at 5 dpi (Figure 2d).

Consistent with these histopathological findings, IHC staining using an anti-MPXV antibody detected viral antigen in lung sections from infected mice. Antigen-positive cells were mainly distributed around bronchiolar and vascular regions and were observed in pneumocytes, alveolar epithelial cells, and pulmonary macrophages. Quantification of A29 antigen staining showed higher H-scores in clade Ib-infected lungs than in clade IIb-infected lungs at the examined time points. The H-score in clade Ib-infected mice increased from 12.92 at 1 dpi to 21.58 at 5 dpi. In clade IIb-infected mice, the H-score was 12.30 at 1 dpi, 15.09 at 5 dpi, and 25.34 at 7 dpi (Table S1).

To exclude the possibility that stronger antigen staining in clade Ib-infected tissues resulted from preferential antibody recognition, Western blot analysis was performed using lysates from clade Ib- and clade IIb-infected cells. The anti-A27 antibody detected A27 protein in both clade Ib- and clade IIb-infected samples, with no signal in the uninfected negative control (Figure S4). These results support the specificity of antibody-based viral antigen detection and suggest that the IHC differences are unlikely to be explained by clade-specific antibody recognition bias. Together, these findings indicate that clade Ib infection is associated with earlier onset and more severe lung pathology than clade IIb infection under the tested intranasal challenge conditions.

Although lung tissue was the major site of productive infection in this model, viral DNA was also detected in upper respiratory tract tissues. To determine whether these signals reflected residual inoculum or productive infection, turbinate and tracheal tissues were collected from mice infected with lethal doses of clade Ib or clade IIb at 1, 3, and 5 dpi. At 1 dpi, viral DNA was undetectable in both tissues from either group. By 3 and 5 dpi, viral DNA was detected in the turbinate and trachea of infected mice, with all samples positive at 5 dpi (Figure S5a, c). Plaque assays confirmed the presence of infectious virus in turbinate tissues, whereas infectious virus in tracheal tissues was detected only in a subset of samples at 3 dpi and was not consistently recovered at 5 dpi (Figure S5b, d). These findings indicate that viral signals in the upper respiratory tract were not solely attributable to residual inoculum.

Respiratory tract viral dynamics were further examined under medium-dose infection conditions. BALB/c mice were infected with clade Ib (5 × 10⁴ PFU) or clade IIb (5 × 10⁵ PFU), and turbinate, trachea, and lung tissues were collected at 1, 3, 5, 7, and 14 dpi. Viral DNA was detected in turbinate tissues from 1 to 7 dpi in both groups, whereas tracheal and lung viral DNA increased at later time points (Figure S6a, c). In contrast, infectious virus recovery was more limited. For clade Ib infection, infectious virus was mainly detected in the lung at 3–7 dpi, with little or no infectious virus recovered from turbinate or tracheal tissues (Figure S6b). For clade IIb infection, infectious virus was detected in turbinate tissue at 1 dpi in a subset of mice and in tracheal tissue at 3 dpi, while lung infectious titers were detected mainly at 3–7 dpi (Figure S6d). By 14 dpi, viral DNA and infectious virus were largely undetectable or markedly reduced in respiratory tissues, consistent with viral clearance at this lower challenge dose.

Together, these data show that both MPXV clade Ib and clade IIb can be detected in upper respiratory tract tissues after intranasal infection. However, infectious virus recovery from the upper respiratory tract was limited compared with lung tissue, supporting the lung as the major site of productive infection in this BALB/c mouse model.

### Pulmonary immune landscape and MPXV cellular distribution following clade Ib and clade IIb infection

**Figure 3.**
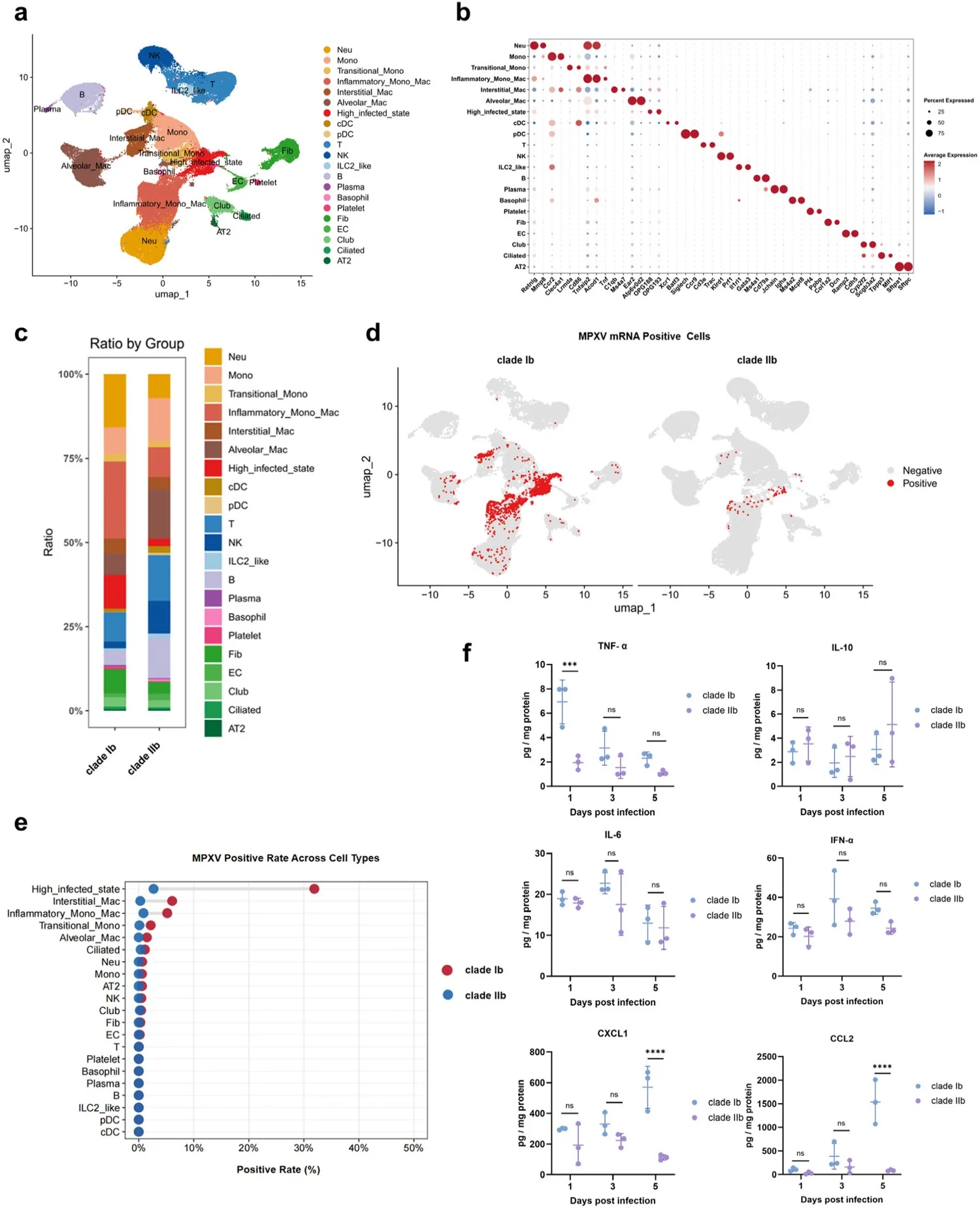
Single-cell transcriptomic analysis and pulmonary cytokine responses in BALB/c mice infected with MPXV clade Ib or clade IIb. BALB/c mice were intranasally infected with MPXV clade Ib (1 × 10⁵ PFU, n = 3) or clade IIb (1 × 10⁶ PFU, n = 3). Lung tissues were collected at 5 dpi for single-cell RNA sequencing. (a) UMAP visualization of integrated single-cell transcriptomic profiles from lung tissues of MPXV clade Ib- and clade IIb-infected mice. Major immune and structural cell populations were identified based on canonical marker gene expression. (b) Dot plot showing representative marker genes used for cell type annotation. Dot size represents the percentage of cells expressing each gene, and colour indicates the average expression level. (c) Relative proportions of major cell populations in lung tissues from clade Ib- and clade IIb-infected groups. (d) UMAP visualization showing the distribution of MPXV mRNA-positive cells in lung tissues from mice infected with clade Ib or clade IIb. Red dots indicate cells in which MPXV transcripts were detected. (e) Comparison of MPXV mRNA-positive cell rates across annotated cell types between the clade Ib- and clade IIb-infected groups. (f) Cytokine and chemokine protein levels in lung homogenates were measured by ELISA at 1, 3, and 5 dpi. Three independent mice were analyzed for each viral clade at each time point, and each point represents one mouse. Concentrations were normalized to total protein content and expressed as pg/mg protein. Data are presented as mean ± SD. Statistical significance was assessed using two-way ANOVA followed by multiple-comparison test. ns, not significant; ***P < 0.001, ****P < 0.0001.

To characterize the pulmonary immune landscape following MPXV infection, single-cell RNA sequencing was performed on lung tissues collected at 5 dpi from BALB/c mice infected with MPXV clade Ib (n = 3) and clade IIb (n = 3). A total of 91,519 cells from the six lung samples were detected. UMAP visualization displayed major immune and structural cell populations, including neutrophils, monocytes, macrophages, dendritic cells, T cells, B cells, NK cells, epithelial cells, endothelial cells, and fibroblasts (Figure 3a and Figure S7). Cell identities were validated by the expression of canonical marker genes (Figure 3b).

The integrated cell composition analysis showed differences between clade Ib- and clade IIb-infected groups, particularly among myeloid populations (Figure 3c and Figure S7). In the combined dataset, inflammatory monocyte/macrophage populations and the high-infected-state cluster accounted for 22.94% and 9.97% of cells from clade Ib-infected lungs, respectively. However, these two populations only made up 8.84% and 2.28% of cells in the clade IIb-infected lungs. Additionally, neutrophils accounted for 15.78% of the cellular population in clade Ib-infected lungs, compared with a much lower proportion of 7.12% in clade IIb-infected lungs. Notably, alveolar macrophages exhibited an opposite distribution pattern: they accounted for 14.56% of cells from clade IIb-infected lungs but merely 6.32% of cells from clade Ib-infected lungs.

We then investigated the distribution of MPXV mRNA-positive cells across cell populations and between viral clades (Figure 3d). MPXV mRNA-positive cells were mainly detected in the high-infected-state cluster, with positivity rates of 31.91% and 2.69% in clade Ib- and clade IIb-infected mice, respectively (Figure 3e). Among myeloid cell subsets in clade Ib-infected lung tissues, interstitial macrophages and inflammatory monocytes/macrophages exhibited MPXV mRNA positivity rates of 6.08% and 5.18%, whereas the corresponding positivity rates in clade IIb-infected lungs were only 0.28% and 0.86%. Furthermore, MPXV mRNA was also detected in alveolar macrophages and monocytes in clade Ib-infected lungs, with positivity rates of 1.48% and 0.59%, respectively. Notably, MPXV mRNA was not detected in the corresponding cell populations from clade IIb-infected lungs (Figure 3d).

To further evaluate cytokine responses following MPXV infection, the transcriptional levels of IFN-α2, IFN-β, TNF-α, IL-6, and IL-10 in lung tissues were analyzed in mice infected with different doses of clade Ib or clade IIb (Figure S8). Compared with control mice, both clade Ib and clade IIb infections resulted in changes in cytokine transcript levels at different time points after infection. At the higher infectious doses (clade Ib: 1 × 10⁵ PFU; clade IIb: 1 × 10⁶ PFU), clade Ib-infected mice showed higher transcript levels of several cytokines, including IFN-α2, IFN-β, TNF-α, and IL-6, at specific time points compared with clade IIb-infected mice, whereas IL-10 expression showed no consistent difference between the two groups (Figure S8).

Cytokine and chemokine protein levels were further measured in lung homogenates by ELISA at 1, 3, and 5 dpi (Figure 3f). Clade Ib-infected mice showed higher levels of TNF-α, CXCL1, and CCL2 at specific time points compared with clade IIb-infected mice, whereas IFN-α, IL-6, and IL-10 showed limited or time-dependent differences between groups. CXCL1 and CCL2 showed higher levels at later time points in clade Ib-infected mice, which coincided with the higher proportions of neutrophils and inflammatory monocyte/macrophage populations observed in the single-cell dataset.

Together, the single-cell and cytokine analyses revealed differences in pulmonary immune profiles between clade Ib- and clade IIb-infected mice. Compared with clade IIb infection, clade Ib infection was associated with differences in pulmonary cell composition, higher detection of MPXV mRNA-positive cells in several myeloid populations, and higher levels of selected cytokines and chemokines at specific time points.

### Differential in vivo therapeutic efficacy of tecovirimat and cidofovir against MPXV clade Ib and clade IIb in BALB/c mice

We first compared the in vitro inhibitory effects of tecovirimat (ST-246) and cidofovir (CDV) against MPXV clades Ib and IIb by quantifying extracellular MPXV DNA levels in culture supernatants using qPCR (Table S2). The half-maximal inhibitory concentration (IC₅₀) values of tecovirimat against clades Ib and IIb were 0.0026 and 0.0012 μM, respectively. For CDV, the IC₅₀ values were determined to be 1.18 and 3.1 μM, respectively.

**Figure 4.**
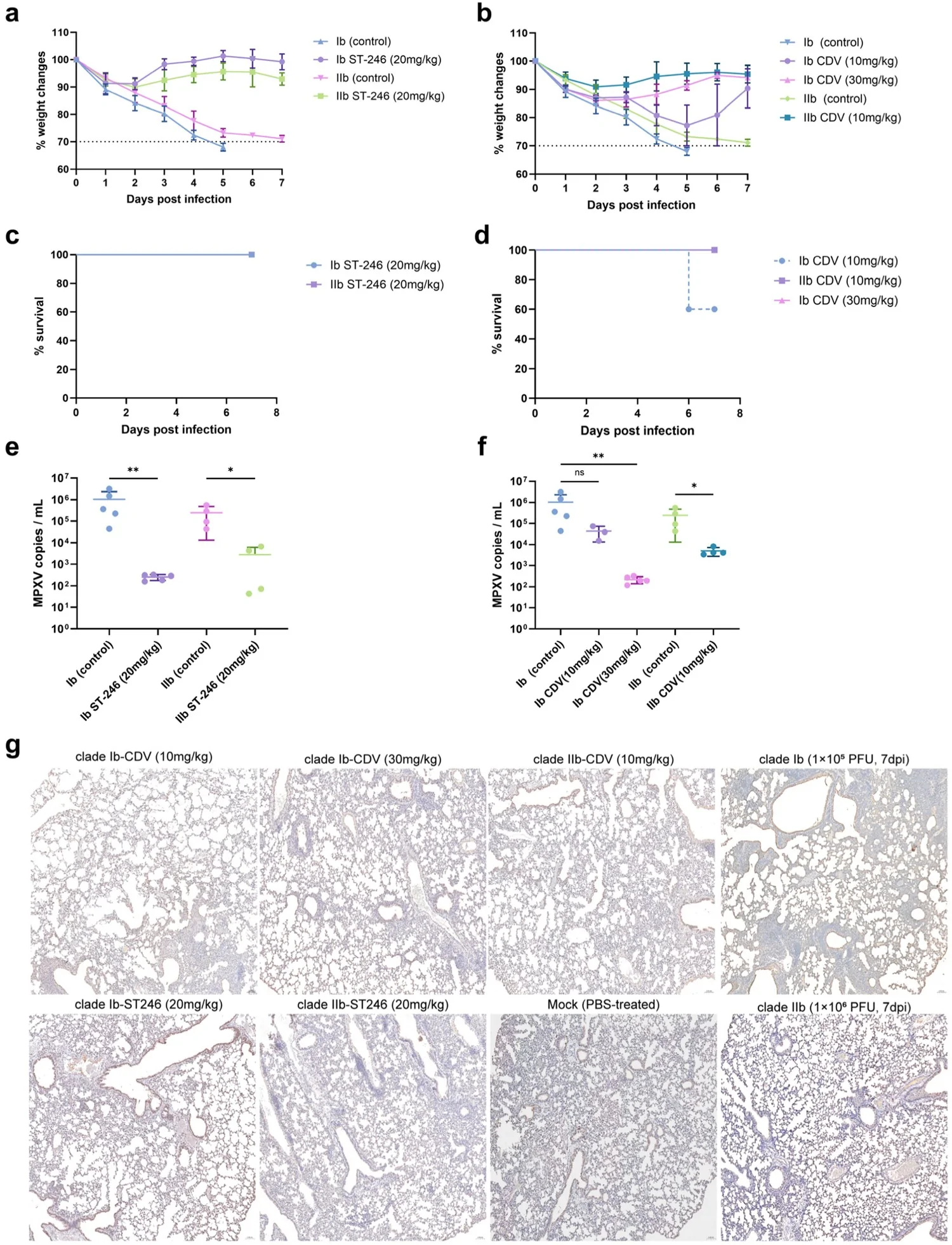
Differential in vivo therapeutic outcomes of cidofovir and tecovirimat against MPXV clade Ib and clade IIb in BALB/c mice. Given the increased pathogenicity of clade Ib in BALB/c mice, we next evaluated the efficacy of antiviral interventions against clade Ib and clade IIb infections. BALB/c mice were intranasally infected with MPXV clade Ib (1 × 10⁵ PFU) or clade IIb (1 × 10⁶ PFU) and then subjected to a 6-day treatment regimen starting from 1 dpi (n = 5). Treatment groups received ST-246 (20 mg/kg, daily oral gavage in 200 μL) or CDV (10/30 mg/kg, daily intraperitoneal injection in 200 μL). (a-d) Daily body weight change (a and b) and survival curves (c and d) of infected mice during treatment. Mice exhibiting >30% weight loss were humanely euthanized according to the approved endpoint criteria. (e, f) Viral genome copies in lung tissues collected from surviving mice at the end of treatment. Data are presented as mean ± SD. *P < 0.05, **P < 0.01 by the Mann–Whitney test. (g) Lung IHC staining of MPXV-infected mice after 6 days of treatment with CDV (10/30 mg/kg) or ST-246 (20 mg/kg). Brown staining indicates viral antigen. Scale bars: 0.5 mm.

Then we evaluated the comparative antiviral efficacy of tecovirimat and cidofovir against clades Ib and IIb in the infection model of BALB/c mice at lethal challenge. Compared to the control group, tecovirimat (20 mg/kg) treated mice began to regain weight from 1 dpi, reaching near baseline by 7 dpi in both clade Ib and IIb infected groups (Figure 4a, c). At 7 dpi, lung viral loads were ∼1 × 10⁶ copies/ml DNA in controls, whereas tecovirimat-treated mice showed markedly reduced viral DNA levels (Figure 4e). Histology revealed that tecovirimat treated mice lungs were largely intact, with minimal perivascular and alveolar lymphocyte/macrophage infiltration, while the control mice displayed interstitial thickening, capillary congestion, perivascular oedema, widespread inflammatory infiltrates, alveolar exudates, haemorrhage, and bronchiolar epithelial hyperplasia (Figure S9). Immunohistochemical staining showed detectable MPXV A29 antigen in the lungs of tecovirimat-treated clade Ib-infected mice. However, antigen levels were markedly reduced compared with untreated controls. To assess viral clearance during tecovirimat treatment, lung viral genome copies and infectious virus titers were measured at serial time points. Both viral DNA levels and infectious virus titers decreased progressively in tecovirimat-treated clade Ib- and clade IIb-infected mice. Together, these results show that tecovirimat markedly reduced pulmonary viral burden compared with untreated controls, supporting its antiviral activity in vivo (Figure S10).

A reference dose of 10 mg/kg cidofovir, reported to provide protective effects in mice was first used [41]. The results showed that two mice in the clade Ib group reached humane endpoints at 6 dpi due to >30% weight loss and severe clinical signs, and the remaining three recovered gradually (Figure 4d). Comparatively, all the mice in the clade IIb group survived and regained weight from 3 dpi (Figure 4b). Moreover, all the mice in the clade Ib group survived and regained weight from 1 dpi when 30 mg/kg of cidofovir was used. Consistent with the weight and survival curve, cidofovir treatment at 10 mg/kg still exhibited high viral loads in the clade Ib group, whereas cidofovir 10 mg/kg for clade IIb and cidofovir 30 mg/kg for clade Ib showed markedly lower viral DNA levels (Figure 4f). Similarly, histology showed preserved lung architecture with mild inflammatory infiltration in the latter two groups, while cidofovir 10 mg/kg-treated clade Ib lungs displayed peribronchiolar and perivascular necrosis, alveolar structure disruption, connective tissue proliferation, and extensive lymphocyte and granulocyte infiltration (Figure S9).

### Discussion

Numerous studies suggest that genomic variations may contribute to differences in virulence and transmissibility between MPXV clades [42]. However, the biological consequences of these variations remain incompletely defined, in part because suitable animal models are still limited, particularly for emerging variants such as clade Ib. Previous work has shown that BALB/c mice are susceptible to MPXV clade IIb infection and can develop lethal disease [39]. In this study, we established an intranasal BALB/c mouse infection model for MPXV clade Ib and found that clade Ib caused lethal disease at an approximately ten-fold lower inoculation dose than clade IIb. Clade Ib viral DNA was detected mainly in respiratory tissues, with low-level detection in the brain and kidney, indicating limited extrapulmonary dissemination under the tested conditions. Clade IIb viral DNA was detected primarily in respiratory tissues. In the lung, clade Ib infection was associated with higher viral burden at later time points, earlier pathological changes, and increased inflammatory mediator responses compared with clade IIb.

The immune background of BALB/c mice should be considered when interpreting these findings. BALB/c mice are known to have a Th2-biased immune tendency and can differ from Th1-prone strains such as C57BL/6 in T cell-mediated and IFN-γ-associated antiviral responses [43,44]. Because T cell immunity and IFN-γ-related responses are important for the control of orthopoxvirus infection [45], the BALB/c model may accentuate certain aspects of disease progression, particularly severe pulmonary inflammation and delayed viral control. Thus, this model is useful for comparing pathogenicity and testing antiviral interventions under a susceptible host background, but it may not fully reproduce immune control mechanisms in other mouse strains or in humans. Future studies using additional genetic backgrounds, together with direct analysis of CD4^+^ and CD8^+^ T cell responses, will be required to define how host immunity shapes clade-specific MPXV pathogenesis.

Consistent with the differential disease progression profiles, clade Ib-infected mice exhibited higher proportions of neutrophils and inflammatory monocyte/macrophage populations than clade IIb-infected mice, as shown by the single-cell RNA-sequencing analysis. Meanwhile, cytokine mRNA analysis and protein measurements provided further evidence of pulmonary inflammation, with significantly elevated protein levels of TNF-α, CXCL1, and CCL2 detected at specific time points in the clade Ib-infected group. Notably, single-cell profiling reflects the relative proportions of immune cell subsets rather than absolute cellular infiltration, and cytokine transcript abundance may not precisely mirror the levels of the corresponding functionally bioactive proteins. Therefore, these inflammatory alterations may not indicate the occurrence of a generalized cytokine storm. Nevertheless, these results demonstrate clade-specific and time-dependent disparities in pulmonary immune cell landscapes and key inflammatory mediators between MPXV clade Ib and clade IIb infections under the tested experimental conditions.

Genomic variation is likely to contribute to the distinct pathogenic profiles of MPXV clades. Comparative genomic analyses have shown approximately 0.5–1.0% genome-wide divergence between clade I and clade II viruses [46,47], with many differences located in regions encoding immune-evasion factors and host-range determinants that are subject to selective pressure [48]. Several immune-regulatory genes differ between clades through substitutions, deletions, or truncations, which may alter viral interactions with host innate immune pathways.

Our data are consistent with the possibility that clade Ib and clade IIb differ in their capacity to shape pulmonary immune responses, although the genetic basis underlying the enhanced virulence of clade Ib remains unresolved. This phenotype is likely to involve the combined effects of multiple genomic variations rather than a single determinant. For example, clade IIb harbours inactivating mutations in several immune-regulatory genes, including OPG204, a B16R homologue, and OPG208, a B19R homologue, which may reduce its ability to suppress antiviral signalling pathways [49]. The continued accumulation of APOBEC3-mediated mutations in clade IIb, including disruptive changes in the IFN antagonist A46R, may further increase viral detectability and facilitate host interferon-mediated control [50,51]. In contrast, clade Ib carries a conserved mutation in OPG125, a D13L homologue, analogous to rifampicin-resistance-associated variants in vaccinia virus, which may have biological consequences during infection [52,53]. Together, these genomic differences may contribute to the divergent pathogenicity observed between clade Ib and clade IIb in vivo. However, functional studies using reverse genetics will be required to determine which mutations directly influence viral replication, tissue tropism, immune modulation, and disease severity.

Currently, tecovirimat is authorized for the treatment of mpox in the United States and Europe, whereas brincidofovir is authorized only in the United States [54]. Tecovirimat inhibits VP37-dependent formation of extracellular enveloped virus and thereby restricts virus spread [54,55]. This finding is consistent with prior evidence showing broad in vitro activity of tecovirimat against MPXV clades Ia, Ib, IIa, and IIb [56]. In our study, tecovirimat showed antiviral activity against both clade Ib and clade IIb. Treatment with tecovirimat at 20 mg/kg reduced body weight loss, lowered pulmonary viral burden, and alleviated lung pathology in both infection groups. Lung viral genome copies and infectious titers declined during treatment, supporting active viral clearance. Residual viral antigen detected by IHC in some treated lungs should not be interpreted as ongoing productive infection by itself, because tecovirimat blocks virion egress rather than viral protein synthesis [57].

The results of cidofovir treatment in our infection model align with earlier reports of cidofovir and brincidofovir showing broad in vitro activity against MPXV clades I, IIa, and IIb [41]. In vitro, cidofovir showed activity against both clade Ib and clade IIb, with IC_50_ values in the same order of magnitude. In vivo, however, cidofovir at 10 mg/kg protected clade IIb-infected mice more effectively than clade Ib-infected mice, whereas increasing the dose to 30 mg/kg improved protection in the clade Ib model. Because the qPCR-based inhibition measurements were within the same order of magnitude between the two clades under the tested conditions, the reduced efficacy of low-dose cidofovir against clade Ib is more likely related to rapid disease progression, higher pulmonary viral burden, and a narrower therapeutic window than to intrinsic cidofovir resistance. A limitation of this study is that antiviral treatment was initiated at 1 dpi. Earlier treatment initiation, delayed-treatment studies, and pharmacodynamic analyses will be needed to define the optimal therapeutic window for clade Ib infection.

In conclusion, intranasal infection of BALB/c mice provides a useful model for comparing MPXV clade Ib and clade IIb pathogenicity and for evaluating antiviral interventions under a susceptible host background. In this model, clade Ib caused lethal disease at a lower inoculation dose, showed limited viral DNA detection outside the respiratory tract, and induced earlier and more severe pulmonary pathology than clade IIb. These findings provide experimental evidence for clade-dependent differences in MPXV pathogenesis and support the use of this model for further studies of viral determinants, host immune responses, and antiviral efficacy. At the same time, the known immune bias of BALB/c mice should be considered when extrapolating these findings to other hosts, and future studies in additional animal models will be important to validate the broader relevance of these observations.

## Materials and methods

### Cells

African green monkey kidney epithelial cells Vero (ATCC CCL-81), human lung carcinoma epithelial cells A549 (ATCC CCL-185), and human embryonic lung fibroblasts MRC-5 (ATCC CCL-171) were maintained at 37°C in a humidified incubator with 5% CO₂. Vero and A549 cells were cultured in Dulbecco’s modified Eagle’s medium (DMEM) supplemented with 10% fetal bovine serum (FBS), 2 mM L-glutamine, 100 U/mL penicillin, 100 μg/mL streptomycin, and 10 mM HEPES. MRC-5 cells were cultured in Minimum Essential Medium (MEM) supplemented with non-essential amino acids (NEAA), 10% FBS, 100 U/mL penicillin and 100 μg/mL streptomycin.

### Viruses

The MPXV clade IIb strain hMpxV/China/SZ-SZTH42/2023/SZTH42 (GISAID accession ID: EPI_ISL_18213374) was isolated from a male mpox patient aged 37 in Shenzhen, China [58]. The clade Ib strain hMpxV/China/GDCDC-468/2024 (National Genomics Data Center accession ID: C_AA113733.1) was isolated from a male mpox patient aged 45. Viruses were grown and titrated in Vero cells. All experiments with live MPXV were performed in an Animal Biosafety Level 3 (ABSL-3) facility following standard biosafety practices.

### Experimental design

Female BALB/c mice (6–8 weeks old) were purchased from GemPharmatech Co., Ltd. (Guangdong branch, China). Upon arrival, mice were acclimatized for 3–5 days prior to experimental procedures and then randomly assigned to experimental groups. On the day of infection, MPXV was diluted in PBS, and mice were anesthetized by intraperitoneal injection of 1.25% tribromoethanol solution at a dose of 0.2 mL/10 g. Anesthesia depth was assessed by respiratory rate and pedal withdrawal reflex to ensure a deep anesthetic state prior to inoculation. Subsequently, mice were intranasally inoculated with 50 μL of virus suspension containing either MPXV clade Ib (2 × 10⁴–1 × 10⁵ PFU, n = 6), MPXV clade IIb (2 × 10⁵–1 × 10⁶ PFU, n = 6), or PBS as a control (n = 6). Surviving animals were euthanized at the designated experimental endpoints. Mice reaching humane endpoints were euthanized immediately according to the approved protocol. Following challenge, mice were monitored at least twice daily for health status and clinical signs. Control animals received the same volume of PBS by the same route. According to ethical guidelines, mice that lost more than 30% of their initial body weight were humanely euthanized.

All MPXV infection studies were conducted under ABSL-3 conditions in individually ventilated cages with ad libitum access to food and water. All experimental protocols were reviewed and approved by the Institutional Animal Care and Use Committee of Shenzhen Third People’s Hospital, and all infectious procedures were carried out under Animal Biosafety Level 3 (ABSL-3) conditions.

### Virus growth curves

A549 and MRC-5 cells seeded in 12-well plates were infected with clade Ib or clade IIb MPXV for 1 h at 37°C at a high multiplicity of infection (MOI = 5) for one-step growth curve analysis or a low MOI (MOI = 0.05) for multi-step growth curve analysis. After virus adsorption, the inoculum was removed, cells were washed with PBS, and fresh culture medium was added. For one-step growth analysis, samples were collected at 48 h post-infection (hpi). For multi-step growth analysis, samples were collected at 0, 24, and 48 hpi. At each time point, culture supernatants were harvested and centrifuged at 2,000 × g for 5 min. The clarified supernatants were collected as extracellular virus (EV) fractions. The pelleted detached cells were combined with infected cells scraped from the culture wells into fresh medium and processed as cell-associated virus (CAV) fractions. All samples were subjected to three freeze–thaw cycles and viral titers were determined by plaque assay on Vero cells in duplicate.

### Virus titration from organs and swab samples

Organ samples collected from infected mice were homogenized in 1 mL of DMEM containing penicillin/streptomycin using a tissue grinder. The homogenate was centrifuged at 3,000 rpm and the supernatant was aliquoted and stored at –80°C for subsequent analysis. Viral titers were determined by plaque assay on Vero cells. Briefly, serial 10-fold dilutions of organ homogenates were inoculated in duplicate into 96-well plates. After 48 h of incubation at 37 °C, cells were fixed with 4% paraformaldehyde and stained as previously described. Viral titers were calculated and expressed as PFU/mL.

For swab samples, each swab was immersed in 1 mL of DMEM supplemented with penicillin/streptomycin and thoroughly vortexed to release viral particles. At the end of the experiment, 200 μL of the sample was used for DNA extraction and subsequent qPCR analysis.

### Quantification of viral DNA

Genomic DNA was extracted from homogenized tissue samples. The viral DNA load of MPXV was quantified using a previously reported qPCR assay with minor adjustments. Specifically, MPXV DNA was isolated from tissue homogenate supernatants using an automated nucleic acid extraction system (Smart 32, Daan Gene) and matched extraction reagents (Daan Gene nucleic acid extraction kit) according to the manufacturer's protocol. Quantitative detection was carried out with the TransScript® Probe One-Step qRT-PCR SuperMix (TransGen Biotech, Beijing) using the following primer set:

Forward: 5′-ACG TGT TAA ACA ATG GGT GAT G-3′

Reverse: 5′-AAC ATT TCC ATG AAT CGT AGT CC-3′

Probe: 5′-FAM-TGA ATG AAT GCG ATA CTG TAT GTG TGG G-BHQ1-3′

### Histopathology and Immunohistochemistry

The middle lobe of the right lung from each mouse was fixed in 10% neutral buffered formalin for 24 h. The tissue was then dehydrated, embedded in paraffin blocks, and sectioned at a thickness of 3 μm. To evaluate pulmonary histopathology, sections were stained with H&E following the manufacturer’s instructions (Servicebio, Wuhan, China). All images were acquired using an optical microscope (Olympus, Tokyo, Japan) and analyzed by pathologists at Servicebio. Image processing was performed using Slide Viewer software.

For immunohistochemical detection of MPXV A29 viral proteins, paraffin-embedded tissue sections were deparaffinized and rehydrated. Antigen retrieval was performed to enhance epitope accessibility, followed by blocking with 5% bovine serum albumin (BSA) to reduce nonspecific binding. Sections were then incubated with a rabbit polyclonal anti-vaccinia virus primary antibody (Abcam, ab35219, 1:200) for detection of the homologous MPXV A29 antigen. Signal detection was performed using a commercial immunohistochemical detection system, according to the manufacturer’s instructions. The reaction was visualized with the IHC Kit D from Servicebio. Stained sections were scanned using the 3DHISTECH system. Positive staining was analyzed via Servicebio's AIpathwell software, following the manufacturer's instructions.

For quantitative analysis, paraffin-embedded lung tissue samples from three mice in each experimental group were used for immunohistochemical evaluation. One lung tissue section from each mouse was subjected to IHC staining, and A29 viral protein expression was evaluated independently for each animal using a modified Histochemistry score [(∑(pi × i) = (percentage of weak intensity cells × 1) + (percentage of moderate intensity cells × 2) + (percentage of strong intensity cells × 3)]. An individual Histochemistry score was obtained for each mouse based on its lung tissue section. The three individual scores in each experimental group were then used to calculate the group mean ± SD (n =   3). The immunohistochemical staining and quantitative analysis were performed by Wuhan Servicebio Technology Co., Ltd. (Hubei, China).

### Western blotting

Vero cells were infected with MPXV clade Ib or clade IIb at an MOI of 0.5. At 48 h post-infection, cells were harvested and lysed in RIPA lysis buffer supplemented with protease and phosphatase inhibitors. Cell lysates were clarified by centrifugation, and the supernatants were separated by SDS–PAGE and transferred onto polyvinylidene difluoride membranes. The membranes were incubated with an anti-VACV A27 antibody (1:2,000 dilution) and an anti-β-actin antibody (1:5,000 dilution). Protein signals were detected using a Bio-Rad ChemiDoc MP Imaging System.

### Single-cell suspension preparation

BALB/c mice were intranasally infected with MPXV clade Ib (1 × 10⁵ PFU, n = 3) or clade IIb (1 × 10⁶ PFU, n = 3). At 5 dpi, lung tissues were collected from each mouse and processed individually without pooling. Each lung sample was rinsed three times with Hank’s balanced salt solution (HBSS) and minced into approximately 1–2 mm fragments for subsequent single-cell preparation and sequencing. The tissue pieces were transferred into 2 mL of GEXSCOPE™ tissue dissociation solution and digested at 37°C for 15 min with intermittent mixing. After digestion, the cell suspension was passed through a sterile 40-μm cell strainer and centrifuged at 1,000 rpm for 5 min. The supernatant was discarded, and the cell pellet was resuspended in 1 mL of PBS.

When excessive red blood cells were present, red blood cell lysis was performed. Briefly, 2 mL of GEXSCOPE™ red blood cell lysis buffer was added to the cell suspension and incubated at 25°C for 10 min. Cells were then centrifuged at 500 × g for 5 min and resuspended in 1 mL of PBS. Cell viability and cell number were determined by trypan blue staining under a light microscope.

### Single-cell RNA library construction and sequencing

Single-cell suspensions stored in PBS were adjusted to a concentration of 1 × 10⁵ cells/mL. The cell suspension was loaded onto a microfluidic chip, and single-cell RNA-seq libraries were prepared using the GEXSCOPE™ Single-Cell RNA Library Kit according to the manufacturer’s instructions. The libraries were diluted to 4 nM and sequenced on an Illumina HiSeq X platform with 150-bp paired-end reads.

### Single-cell RNA-seq data processing and analysis

Raw sequencing data were processed using the Singleron internal analysis pipeline to generate gene expression matrices. Briefly, Read 1 sequences without poly-T stretches were removed, and valid cell barcodes and unique molecular identifiers (UMIs) were extracted. Read 2 sequences were trimmed to remove adapters and poly-A tails using fastp v1. Reads were then aligned to the mouse reference genome (GRCm39) from the Ensembl database, followed by gene quantification using STAR v2.5.3a and featureCounts v1.6.2. Reads sharing the same cell barcode, UMI, and gene assignment were grouped, and UMI counts for each gene in each cell were used to construct the expression matrix. Additionally, to detect and quantify viral transcripts, reads were mapped to specific MPXV reference genomes: NCBI accession PQ178862.2 for the clade Ib infected samples, and NGDC accession C_AA061241.1 for the clade IIb-infected samples. Finally, to accurately identify MPXV mRNA-positive cells and eliminate ambient viral RNA contamination, a conditional two-stage Gaussian mixture model (GMM) was fitted based on the percentage of cellular viral RNA.

Downstream analysis was performed using the Seurat v5 package in R [59]. Quality control removed cells with fewer than 200 genes and more than 10% mitochondrial reads, as well as genes detected in fewer than three cells. Doublet detection and removal were performed with scDblFinder [60], and after removal of doublets, 91,519 cells were retained for downstream analysis. Data were normalized and scaled using the SCTransform method with 3,000 variable genes, followed by batch correction with Harmony v2 applied to the principal component (PC) space based on the top 30 PCs (of 50) [61]. Graph-based clustering was carried out with the FindClusters function at a resolution of 0.4. Differential expression between clusters was assessed using the FindAllMarkers function with Wilcoxon rank-sum test and an adjusted P-value threshold of 0.05 (Benjamini-Hochberg correction). Cell identities were assigned by matching cluster-specific markers to established canonical markers for murine lung cell populations [62].

### ELISA

BALB/c mice were intranasally infected with MPXV clade Ib at 1 × 10⁵ PFU or MPXV clade IIb at 1 × 10⁶ PFU. For each infection group and each time point, three mice were used (n = 3 per group per time point). Lung tissues were collected at 1, 3, and 5 dpi, and cytokine and chemokine levels in lung homogenates were measured by ELISA according to the manufacturers’ instructions. The following analytes were quantified: IFN-α (Cloud-Clone Corp., SEA033Mu), TNF-α (Lianke Biotech, EK282HS), IL-10 (Cloud-Clone Corp., SEA056Mu), IL-6 (Cloud-Clone Corp., SEA079Mu), CXCL1 (Cloud-Clone Corp., SEA041Mu), and CCL2 (Invitrogen, 88-7391). Total protein concentrations in lung homogenates were determined using a BCA Protein Assay Kit (Servicebio, G2026). Cytokine and chemokine concentrations were normalized to total protein content and expressed as pg/mg protein.

### Cytokine profile

BALB/c mice were infected with MPXV clade Ib or clade IIb at lethal doses of 1 × 10⁵ PFU (clade Ib) and 1 × 10⁶ PFU (clade IIb), or at non-lethal doses of 5 × 10⁴ PFU (clade Ib) and 5 × 10⁵ PFU (clade IIb). At 1, 3, and 5 dpi, lung tissues were collected from mice in each dose group at each time point (n = 3 mice per dose group per time point). Total RNA was extracted from 200 µL of lung tissue homogenate supernatant from each individual mouse using TRIzol reagent (Solarbio, Beijing, China). Reverse transcription and quantitative PCR were performed using the HiScript II One Step qRT-PCR SYBR Green Kit (Vazyme, Q221-01). Primers were designed based on published references [49]. The thermocycling parameters were as follows: 55°C for 5 min, 95°C for 30 s, followed by 40 cycles of 95°C for 10 s and 60°C for 45 s. Data analysis was performed using CFX Maestro 2.3 software (Bio-Rad), and relative gene expression was calculated using the 2^−ΔΔ^ CT method[63].

### Assessment of the antiviral efficacy of cidofovir and tecovirimat against MPXV

The antiviral effects of cidofovir and tecovirimat were evaluated by quantifying extracellular MPXV DNA in culture supernatants using qPCR. Vero cells were seeded into 96-well plates and cultured overnight before infection with MPXV at an MOI of 0.1, as previously described for orthopoxvirus antiviral evaluation studies [64]. Following a 1-h adsorption period, the inoculum was removed, and the cells were washed three times with PBS. Maintenance medium containing serial dilutions of cidofovir or tecovirimat was then added to the wells at 100 μL per well. Cidofovir was prepared at an initial concentration of 100 μM, and tecovirimat was prepared at an initial concentration of 100 nM, followed by three-fold serial dilutions. Each concentration was tested in three replicate wells. At 72 h post-infection, culture supernatants from the three replicate wells were pooled, and 200 μL of the pooled samples was used for nucleic acid extraction and subsequent qPCR analysis.

BALB/c mice were infected with MPXV clade Ib (1 × 10⁵ PFU) or clade IIb (1 × 10⁶ PFU), with 4–6 mice included per infection group. For therapeutic studies, treatment was initiated at 1 dpi. Mice received tecovirimat orally at 20 mg/kg or cidofovir intraperitoneally at 10 or 30 mg/kg once daily for 6 consecutive days, according to the assigned treatment group. Each dose was administered in a volume of 200 μL. Control mice received the corresponding vehicle via the same route and schedule. At 7 dpi, animals were euthanized, and lung tissues were collected for viral titer determination and histopathological analysis.

For viral kinetics analysis of tecovirimat treatment, BALB/c mice were infected with MPXV clade Ib (1 × 10⁵ PFU) or clade IIb (1 × 10⁶ PFU). Tecovirimat treatment was initiated at 1 dpi and administered orally once daily at 20 mg/kg (200 μL per dose). Mice were euthanized at 2, 4, and 6 dpi, and lung tissues were collected for viral analysis. Three mice were included in each treatment group at each time point (n = 3 per group per time point). Pulmonary viral genome copies were quantified by qPCR, and infectious virus titers were determined by plaque assay. Statistical analyses were performed as described in the figure legends.

### Statistics

All data analyses were performed with GraphPad Prism version 10.4 (GraphPad Software). Statistical tests were selected according to the experimental design and are indicated in the corresponding figure legends.

## Supplementary Material

Supplementary information.doc

Supplementary Tables S2.docx

Supplementary Tables S1.docx

## Data Availability

The authors confirm that all data generated and analyzed during this study are either included in this published article or available from the corresponding authors upon reasonable request.
