# Supplementary material for: Differential pathogenesis and antiviral treatment outcomes of MPXV clade Ib and clade IIb in a BALB/c mouse model
Source: Emerg Microbes Infect. 2026 Sep 16;15(1):2724633. doi: 10.1080/22221751.2026.2724633 (PMC13587567; doi:10.1080/22221751.2026.2724633)
Supplement: Supplementary file 1 [file TEMI_A_2724633_SM2873.docx]

**Supplementary Table 1. Modified histochemistry scores of A29 viral protein expression in the lungs of BALB/c mice infected with MPXV clade Ib/IIb**

|  | days post infection  (dpi) | Positive Area, % | Histochemistry score  (H-Score) |
| --- | --- | --- | --- |
|  | 1 | 9.43 ± 2.65 % | 12.92 ± 3.45 |
| clade Ib (1 × 10^5^ PFU) | 3 | 10.52 ± 1.39 % | 14.47 ± 2.57 |
|  | 5 | 13.91 ± 1.39 % | 21.58 ± 3.65 |
|  | 7 | - | - |
|  | 1 | 8.32 ± 1.21 % | 12.3 ± 1.79 |
| clade IIb (1 × 10^6^ PFU) | 3 | 9.34 ± 1.42 % | 13.51 ± 3.12 |
|  | 5 | 10.42 ± 1.30 % | 15.09 ± 2.78 |
|  | 7 | 15.56 ± 4.56 % | 25.34 ± 9.27 |
| clade Ib CDV (10 mg/kg) | 7 | 14.32 ± 3.93 % | 20.25 ± 5.69 |
| clade Ib CDV (30 mg/kg) | 7 | 6.28 ± 1.63 % | 8.03 ± 2.46 |
| clade IIb CDV (10 mg/kg) | 7 | 11.6 ± 0.48 % | 15.62 ± 1.29 |
| clade Ib ST-246 (20 mg/kg) | 7 | 8.66 ± 4.14 % | 12.39 ± 5.24 |
| clade IIb ST-246 (20 mg/kg) | 7 | 6.42 ± 0.74 % | 7.72 ± 1.72 |
| Control | - | 4.19 ± 1.57 % | 8.33 ± 1.66 |

Note: Data are presented as mean ± SD; “-”, no sample.
