# Supplementary material for: Differential pathogenesis and antiviral treatment outcomes of MPXV clade Ib and clade IIb in a BALB/c mouse model
Source: Emerg Microbes Infect. 2026 Sep 16;15(1):2724633. doi: 10.1080/22221751.2026.2724633 (PMC13587567; doi:10.1080/22221751.2026.2724633)
Supplement: Supplementary file 2 [file TEMI_A_2724633_SM2874.docx]

**Supplementary Table 2. qPCR-based inhibition of extracellular MPXV DNA by cidofovir and tecovirimat**

| Drug name | MPXV Clade Ib  qPCR-based IC₅₀ (μM) | MPXV Clade IIb  qPCR-based IC₅₀ (μM) |
| --- | --- | --- |
| Cidofovir (CDV) | 1.18 ± 0.25 | 3.1 ± 0.49 |
| Tecovirimat (ST-246) | 0.0026 ± 0.0004 | 0.0012 ± 0.0003 |

Note: Data are presented as mean ± SD. IC₅₀ values were derived from dose–response curves based on the reduction in extracellular MPXV DNA copies in culture supernatants, as quantified by qPCR at 72 h post-infection.
