# Supplementary material for: Differential pathogenesis and antiviral treatment outcomes of MPXV clade Ib and clade IIb in a BALB/c mouse model
Source: Emerg Microbes Infect. 2026 Sep 16;15(1):2724633. doi: 10.1080/22221751.2026.2724633 (PMC13587567; doi:10.1080/22221751.2026.2724633)
Supplement: Supplementary file 3 [file TEMI_A_2724633_SM2875.doc]

# S1: Intranasal infection of BALB/c mice with MPXV clade Ib results in lethal outcome.

Groups of BALB/c mice (n = 6) were inoculated intranasally with MPXV clade Ib (2 × 10⁵ PFU in 50 μL per mouse) and daily weight loss (a) and survival (b) were monitored daily for 5 days. At 5 dpi, mice reaching the humane endpoint of >30% body weight loss were euthanized according to the approved protocol. Data are presented as the mean ± SD. Statistical significance was determined by unpaired Student’s t-tests. **P < 0.01, ****P < 0.0001.

# S2: Detection of MPXV in the oropharyngeal swabs of BALB/c mice with intranasal infection.

Oropharyngeal swabs were collected from mice infected with MPXV clade Ib (1 × 10⁵ PFU) or clade IIb (1 × 10⁶ PFU) at 1, 3, and 5 dpi. Numbers above the scatter diagram denote the positivity rate of samples. Data are presented as the mean ± SD. Statistical significance was assessed by two-way ANOVA followed by Dunnett’s multiple-comparisons test. ****P < 0.0001.

**S3: Comparison of cell-associated and extracellular virus production by clade Ib and clade IIb MPXV in A549 and MRC-5 cells.**

A549 human pulmonary epithelial cells and MRC-5 human lung fibroblasts were infected with clade Ib or clade IIb MPXV. For one-step growth analysis (a, b), cells were infected at an MOI of 5 and harvested at 48 h post-infection for quantification of cell-associated virus (CAV) and extracellular virus (EV). For multi-step growth analysis (c, d, e, f), cells were infected at an MOI of 0.05, and CAV and EV titers were measured at 0, 24, and 48 h post-infection. Viral titers were determined by plaque assay and expressed as PFU/mL. Data represent mean ± SD of three independent experiments. ns, not significant; *P < 0.05 using an unpaired t-test.

**S4: Validation of anti-A27 antibody reactivity against MPXV clade Ib and clade IIb.**

Cell lysates from MPXV clade Ib- and clade IIb-infected Vero cells were subjected to Western blot analysis using an anti-A27 antibody. β-actin served as a loading control. NC, uninfected negative control.

**S5: Viral loads in turbinate and tracheal tissues following MPXV clade Ib and clade IIb infection.**

Turbinates and tracheal tissues were collected from mice infected with MPXV clade Ib (1 × 10⁵ PFU) or clade IIb (1 × 10⁶ PFU) at 1, 3, and 5 dpi. (a) Viral genome copy numbers in turbinate tissue determined by qPCR. (b) Infectious virus titers in turbinate tissue determined by plaque assay. (c) Viral genome copy numbers in tracheal tissue determined by qPCR. (d) Infectious virus titers in tracheal tissue determined by plaque assay. Numbers above the scatter plots indicate the proportion of positive samples. Data are presented as the mean ± SD. Statistical significance was assessed by two-way ANOVA followed by Dunnett’s multiple-comparisons test. ns, not significant; **P < 0.01, ***P < 0.001, ****P < 0.0001.

# S6: Genome copies and infectious virus of MPXV clade Ib and clade IIb in the respiratory tract of BALB/c mice within 14 days post infection.

(a-d) BALB/c mice (n = 6) were intranasally infected with MPXV clade Ib (5 × 10^4^ PFU) or clade IIb (5 × 10^5^ PFU). Turbinates, trachea, and lung tissues were collected at 1, 3, 5, 7, and 14 dpi for quantification of viral genome copies by qPCR (a and c) and infectious virus via plaque assay (b and d). Data are presented as the mean ± SD. Statistical significance was assessed by two-way ANOVA followed by Dunnett’s multiple-comparisons test. ns, not significant; *P < 0.05, **P < 0.01, ***P < 0.001, ****P < 0.0001.

# S7: Single-cell transcriptomic profiles and cell composition across individual lung samples from MPXV-infected BALB/c mice.

Lung tissues were collected from six BALB/c mice at 5 dpi, including three mice infected with MPXV clade Ib (1 × 10⁵ PFU) and three mice infected with MPXV clade IIb (1 × 10⁶ PFU). (a) UMAP plots showing the distribution of annotated cell populations in individual lung samples. Cells are colored according to cell type annotations. (b) Relative abundance of annotated cell populations in each individual lung sample, shown as stacked bar plots. Each bar represents one individual mouse lung sample. The same color scheme is used for cell populations in panels (a) and (b). (c) UMAP plots showing the distribution of MPXV mRNA-positive cells in individual lung samples. Red dots indicate cells detected with MPXV transcripts, while gray dots indicate MPXV mRNA-negative cells.

# S8: Pulmonary cytokine transcriptional responses following low- and high-dose MPXV clade Ib and clade IIb infections in BALB/c mice.

BALB/c mice were infected with MPXV clade Ib (a) or clade IIb (b) at the indicated lethal (clade Ib: 1 × 10⁵ PFU; clade IIb: 1 × 10⁶ PFU) or non-lethal doses (clade Ib: 5 × 10⁴ PFU; clade IIb: 5 × 10⁵ PFU), with n = 3 mice per dose group. (c) Direct comparison of cytokine transcript levels between mice infected with the higher doses of MPXV clade Ib (1 × 10⁵ PFU) and clade IIb (1 × 10⁶ PFU). RNA was extracted from lung tissues, and the transcript levels of IFN-α2, IFN-β, IL-6, TNF-α, and IL-10 were measured. The data were normalized to β-actin. Data are presented as mean ± SD. Statistical significance was assessed by two-way ANOVA followed by multiple-comparisons test. ns, not significant; *p < 0.05, **p < 0.01, ***p < 0.001.

# S9: Lung histopathology after cidofovir or ST-246 treatment in MPXV-infected BALB/c mice.

H&E staining of lung sections from MPXV clade Ib (1 × 10⁵ PFU) or clade IIb (1 × 10⁶ PFU) infected mice after a 6-day treatment with cidofovir (10/30 mg/kg) or ST-246 (20 mg/kg). Black arrow: Lymphocytic and granulocytic infiltration. Light blue arrow: Necrotic cellular debris. Blue arrow: hemorrhage. Scale bars: 1 mm (overview), 100 μm (magnified view).

**S10: Effects of ST-246 treatment on pulmonary viral genome loads and infectious virus titers following MPXV infection.**

BALB/c mice were infected with MPXV clade Ib (1 × 10⁵ PFU) or clade IIb (1 × 10⁶ PFU). ST-246 treatment was initiated at 1 dpi and administered orally once daily at 20 mg/kg. Lung tissues were collected at 2, 4, and 6 dpi for viral analysis. Three mice were included in each group at each time point (n = 3). (a) Pulmonary viral genome copies in clade Ib-infected mice determined by qPCR. (b) Pulmonary infectious virus titers in clade Ib-infected mice determined by plaque assay. (c) Pulmonary viral genome copies in clade IIb-infected mice determined by qPCR. (d) Pulmonary infectious virus titers in clade IIb-infected mice determined by plaque assay. Data are presented as mean ± SD. Statistical significance was assessed by two-way ANOVA followed by Dunnett’s multiple-comparisons test. ns, not significant; ****P < 0.0001.
